# Herbarium collection of the Rio de Janeiro Botanical Garden (RB), Brazil

**DOI:** 10.3897/BDJ.6.e22757

**Published:** 2018-03-12

**Authors:** João M. Lanna, Luís Alexandre E da Silva, Marli P. Morim, Paula M. Leitman, Natália O. Queiroz, Fabiana L. R. Filardi, Eduardo C. Dalcin, Felipe A. Oliveira, Rafaela C. Forzza

**Affiliations:** 1 Rio de Janeiro Botanical Garden, Rio de Janeiro, Brazil

**Keywords:** Southern Brazil, Atlantic forest, Amazon forest, neotropical flora, taxonomy

## Abstract

**Background:**

This paper provides a quantitative and general description of the Rio de Janeiro Botanical Garden herbarium (RB) dataset. Created over a century ago, the RB currently comprises ca. 750,000 mounted specimens, with a strong representation of Brazilian flora, mainly from the Atlantic and Amazon forests. Nearly 100% of these specimens have been entered into the database and imaged and, at present, about 17% have been geo-referenced. This data paper is focused exclusively on RB's exsiccatae collection of land plants and algae, which is currently increasing by about twenty to thirty thousand specimens per year thanks to fieldwork, exchange and donations. Since 2005, many national and international projects have been implemented, improving the quality and accessibility of the collection. The most important facilitating factor in this process was the creation of the institutional system for plants collection and management, named JABOT. Since the RB is continuously growing, the dataset is updated weekly on SiBBr and GBIF portals.

**New information:**

The most represented environments are the Atlantic and Amazon forests, a biodiversity hotspot and the world's largest rain forest, respectively. The dataset described in this article contains the data and metadata of plants and algae specimens in the RB collection and the link to access the respective images. Currently, the RB data is publicly available online at several biodiversity portals, such as our institutional database JABOT, the Reflora Virtual Herbarium, the SiBBr and the GBIF portal. However, a description of the RB dataset as a whole is not available in the literature.

## Introduction

Created in 1890, the RB herbarium of the Rio de Janeiro Botanical Garden (JBRJ) is composed of seven botanical collections consisting of: mounted specimens (RB – 750,000, with 7,500 nomenclatural types and around 3,000 paratypes), wood (RBw - ca. 10,300 specimens), fruits (RBcarpo - ca. 8,000 specimens), DNA bank (RBdna - ca. 5,700 specimens), spirit (RBspirit - ca. 2,500 specimens), seed bank (RBsem - ca. 2,700 specimens) and ethnobotany (RBetno – ca. 200 specimens). For further details about the history and structure of the herbarium see (Marquete et al. 2001, Forzza et al. 2015b, Forzza et al. 2016). This data paper is focused on the main mounted plants and algae herbarium specimens, which are currently increasing by about twenty to thirty thousand new specimens per year.

Samples are organised alphabetically across two floors in the herbarium building: the dicots families of angiosperms from A to N are stored on the first floor; the remaining families of dicots, monocots, gymnosperms, ferns, lycophytes, bryophytes, algae, fungi, lichens and the other collections (fruits, spirit, wood and ethnobotanical) on the second floor.

Regarding the adopted taxonomic classification system, due to its large size and the efforts involved in rearranging so many specimens, currently the angiosperms are organised according to Cronquist (1981) and are gradually being reorganised to APG IV (2016). The gymnosperms are organised according to Christenhusz et al. (2011); ferns and lycophytes follow PPG I (2016); bryophytes follow Söderström et al. (2016); and algae follow Guiry and Guiry (2017).

**Figure 1.**
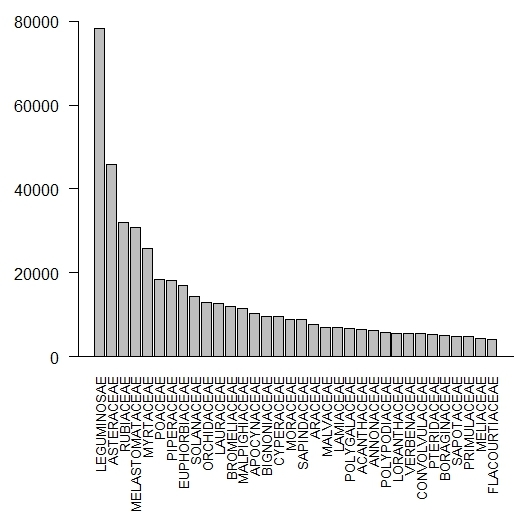
Number of specimens per botanical family (≥ 4.000 specimens) deposited in the RB herbarium.

Fig. 1 shows the families that have ≥4,000 specimens deposited in RB. Amongst these 34 families, 32 belong to angiosperms and two to ferns. The four angiosperm families with the highest amount of specimens are: Fabaceae (78,437), Asteraceae (45,871), Rubiaceae (31,971) and Melastomataceae (30,907). Non-angiosperm groups are represented as follows: Gymnosperms - Podocarpaceae (187), Gnetaceae (112), Pinaceae (73) and Araucariaceae (59); Ferns - Polypodiaceae (5,733), Pteridaceae (5,280), Dryopteridaceae (3,708) and Aspleniaceae (2,532); Bryophyte - Lejeuneaceae (2,439), Sphagnaceae (707), Leucobryaceae (622) and Metzgeriaceae (613); Algae - Rhodomelaceae (1,334), Cladophoraceae (835), Dictyotaceae (620) and Corallinaceae (487).

**Figure 2.**
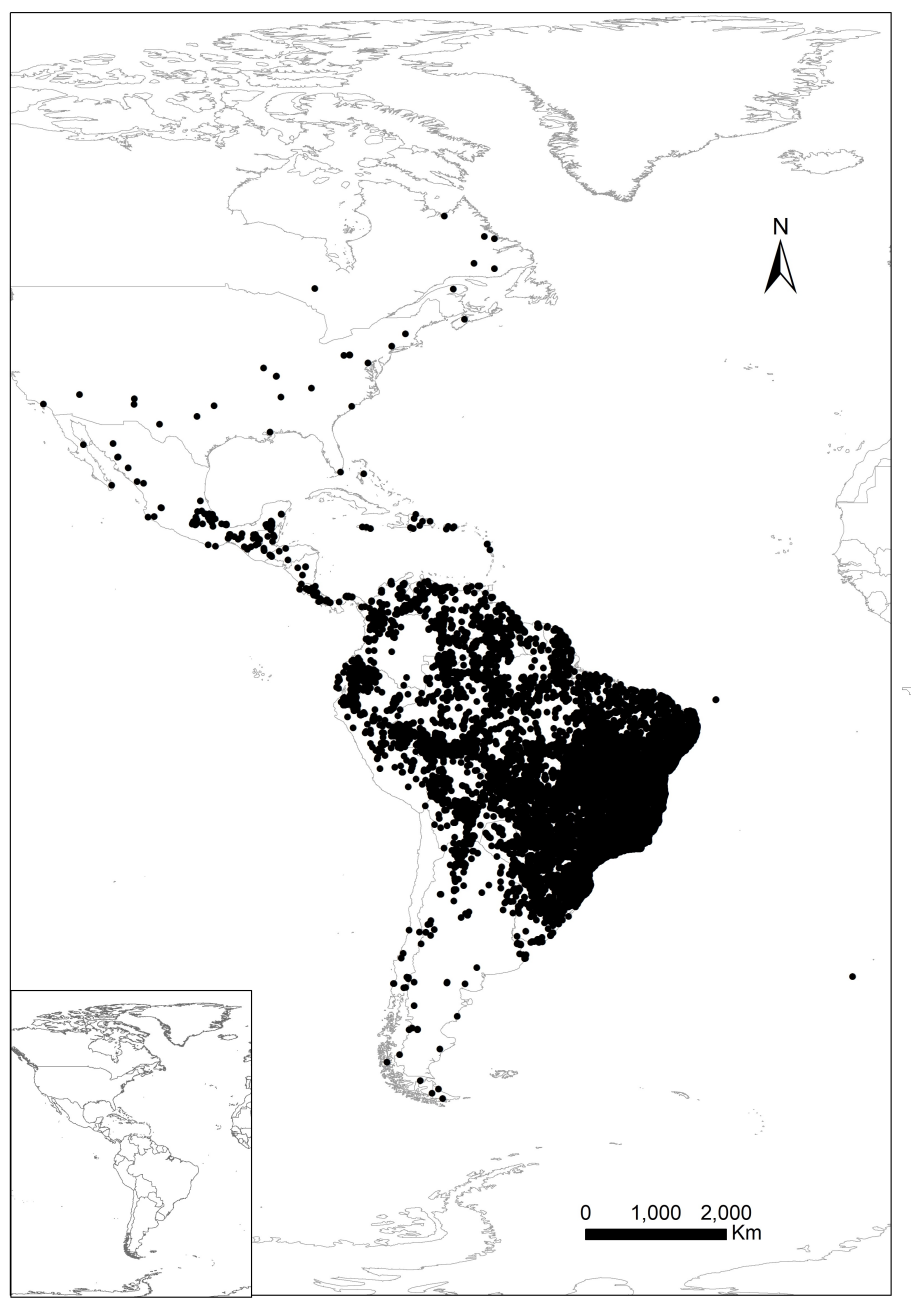
Georeferenced specimen deposited in the RB herbarium (ca. 17% of the total).

The collection is completely digitised and it is managed using the JABOT system (Silva et al. 2017, Gonzales 2009), which was developed by JBRJ staff. With the implementation of the REFLORA project (Forzza et al. 2015a, Nic Lughadha et al. 2016), nearly 100% of the mounted specimens were photographed and are available through the public interface of the JABOT system and in other biodiversity portals, such as the Reflora Virtual Herbarium (Forzza et al. 2015a), the Brazilian Biodiversity Information System (SiBBr) (Gadelha et al. 2014) and the Global Biodiversity Information Facility (GBIF) (Robertson et al. 2014). Currently, new accessions are digitised and photographed before they are incorporated into the herbarium and all new identifications are updated in the JABOT system on a daily basis. At present, about 17% of the specimens have been assigned geographic coordinates (Fig. 2).

### Temporal coverage

**Figure 3.**
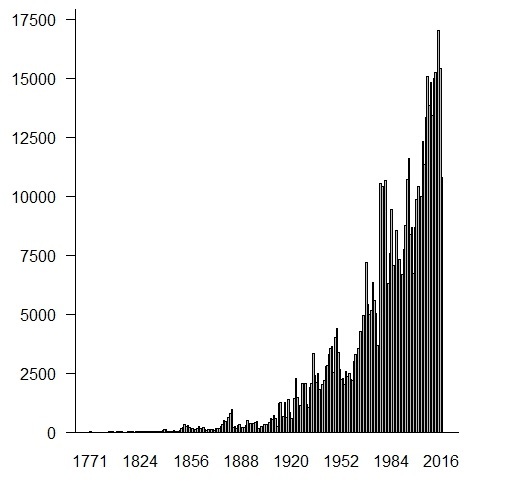
Number of specimens deposited in the RB herbarium by year of collection.

The RB herbarium was created by the naturalist João Barbosa Rodrigues in 1890. The first specimens came from a private collection of about 25,000 specimens donated by Emperor Dom Pedro II (Marquete et al. 2001). Aiming to make the herbarium a reference for the study of the Brazilian flora, Barbosa Rodrigues and other naturalists (e.g. A. Ducke, J.G. Kuhlmann, A.P. Duarte, A.C. Brade, D. Sucre) conducted many expeditions throughout Brazil, considerably enriching the collection. The herbarium has been a national reference since then, increasing its collections over the years (Fig. 3).

## Sampling methods

### Sampling description

The herbarium specimen collection combines specimens from institutional projects, undergraduate and post-graduate research and exchanges or donations from other herbaria. In addition, from 1970 onwards, relevant national projects of flora documentation sent specimens to RB, such as RADAMBRASIL Project (1970-1985) and the Flora Program CNPq (1975-1983, MCT/CNPq 1987). Furthermore, as the official custodian for the Ministry of Environment, RB also receives many specimens from private companies with activities related to environmental impact studies and phytochemical products and most of these specimens are donated in exchange for identification. The institution includes 53 associate researchers and hosts around 550 visiting taxonomists every year, standing out as the most visited herbarium in Brazil.

### Quality control

The RB uses the institutional system JABOT to perform all functions regarding herbarium management (i.e. loans, donations, new specimen registration, database management, quality control and web publication). The JABOT is a PostgreSQL database management system with 117 tables specifically created for botanical collections. The data insertion can be made directly into the JABOT interface or via uploading spreadsheets, with controlled and free text fields (Silva et al. 2017).

### Step description

**Plant processing procedures**

The herbarium follows the usual procedures for processing specimens (Bridson and Forman 2000, Simpson 2006). Fresh materials are pressed and dried over a stove or in an oven. Once they are dry, specimens are glued on to acid-free paper, with gummed cloth tape or thread for bulky plants. Bryophytes, fungi and lichens are placed into acid-free packets. Algae can also be mounted on acid-free paper with gummed cloth tape or stored in plastic boxes in the case of calcareous algae.

**Collection digitisation history**

**Figure 4.**
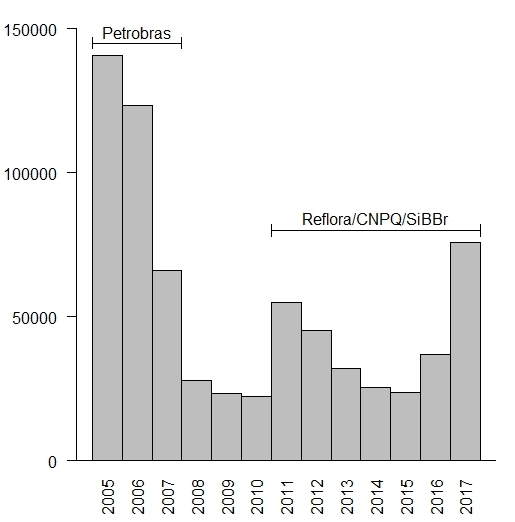
Number of specimens databased in JABOT system per year.

Once JABOT was created in 2005, data entry for the herbarium started with the “*Projeto de Informatização*” funded by Petrobras, which lasted until 2007. After this, between 2008 and 2010, without a project specifically directed towards data inclusion into the system, this task was performed only for new specimens of previously digitised families, by a smaller team, part of institutional projects and core herbarium staff. At the end of 2010, the Reflora project started, boosting data entry into the system and was completed during 2014. The GFJP and GUA herbaria were incorporated into RB in 2016 and 2017, respectively, which substantially increased the number of specimens entered into the system, as can be seen in Fig. 4.

## Geographic coverage

### Description

**Figure 5.**
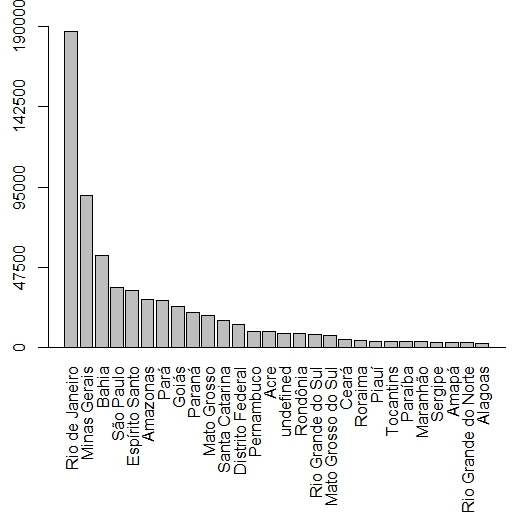
Number of vouchers per Brazilian states deposited in the RB herbarium.

The majority of specimens were collected in Brazil (ca. 90%) and the country’s most widely represented region is the Southeast, where the herbarium is based (ca. 349.000 specimens, 50% of the total). The south-eastern states of Rio de Janeiro (ca. 189,000 specimens) and Minas Gerais (ca. 90,000) are represented by the largest number of specimens (Fig. 5). It should be noted that most of this region is part of the Atlantic Forest and Rio de Janeiro state is positioned entirely within this biome.

North Brazil ranks second in number of specimens and the states of Amazonas and Pará are the best represented, with ca. 29,000 (ca. 4%) and 28,000 (ca. 4%), respectively (Fig. 5). One of the first great plant collectors in the region, especially with regard to Amazonian flora, was Adolf Ducke, who conducted expeditions in the states of Amazonas and Pará, mainly in the first half of the 20th century.

### Coordinates

-34.1618 and 5.5285 Latitude; -34.4531 and -74.1796 Longitude.

## Taxonomic coverage

### Description

Taxonomic ranks

**Group**: Spermatophyta

**Family**: ACANTHACEAE (6603 specimens/773 names); ACERACEAE (52/43); ACHARIACEAE (96/19); ACHATOCARPACEAE (27/4); ACORACEAE (3/6); ACTINIDIACEAE (33/19); ADOXACEAE (14/3); AGAVACEAE (82/43); AIZOACEAE (84/20); ALISMATACEAE (504/64); ALSTROEMERIACEAE (489/47); AMARANTHACEAE (2664/273); AMARYLLIDACEAE (727/220); ANACARDIACEAE (2918/227); ANISOPHYLLEACEAE (20/6); ANNONACEAE (6334/548); ANTHERICACEAE (18/10); APIACEAE (923/144); APOCYNACEAE (10292/1144); APODANTHACEAE (12/5); APONOGETONACEAE (2/2); AQUIFOLIACEAE (1382/168); ARACEAE (7726/1102); ARALIACEAE (1957/499); ARAUCARIACEAE (59/17); ARECACEAE (2235/546); ARISTOLOCHIACEAE (693/166); ASCLEPIADACEAE (3520/543); ASPARAGACEAE (272/115); ASPHODELACEAE (6/6); ASTERACEAE (45873/5672); ASTERANTHACEAE (5/1); AVICENNIACEAE (129/12); BALANOPHORACEAE (230/22); BALSAMINACEAE (61/13); BARRINGTONIACEAE (9/7); BASELLACEAE (64/8); BATACEAE (5/1); BEGONIACEAE (3434/251); BERBERIDACEAE (149/42); BETULACEAE (86/52); BIGNONIACEAE (9683/968); BIXACEAE (250/14); BOMBACACEAE (1465/187); BONNETIACEAE (142/17); BORAGINACEAE (5052/419); BRASSICACEAE (159/97); BROMELIACEAE (12391/1641); BURMANNIACEAE (224/49); BURSERACEAE (2006/171); BUTOMACEAE (7/2); BUXACEAE (13/6); CABOMBACEAE (59/10); CACTACEAE (3026/351); CALCEOLARIACEAE (6/3); CALLITRICHACEAE (15/7); CALOPHYLLACEAE (55/7); CALYCANTHACEAE (3/2); CALYCERACEAE (107/16); CAMPANULACEAE (1410/184); CANELLACEAE (51/12); CANNABACEAE (266/17); CANNACEAE (245/18); CAPPARACEAE (975/91); CAPRIFOLIACEAE (258/89); CARDIOPTERIDACEAE (22/7); CARICACEAE (160/22); CARYOCARACEAE (312/26); CARYOPHYLLACEAE (1156/468); CASUARINACEAE (41/12); CECROPIACEAE (110/22); CELASTRACEAE (2542/209); CENTROLEPIDACEAE (4/3); CERATOPHYLLACEAE (14/5); CHENOPODIACEAE (309/118); CHLORANTHACEAE (235/22); CHRYSOBALANACEAE (3841/339); CISTACEAE (60/32); CLEOMACEAE (513/56); CLETHRACEAE (346/19); CLUSIACEAE (2439/264); COCHLOSPERMACEAE (131/9); COLCHICACEAE (20/13); COMBRETACEAE (2715/180); COMMELINACEAE (2492/146); CONNARACEAE (1035/115); CONVALLARIACEAE (44/23); CONVOLVULACEAE (5549/504); CORIARIACEAE (11/6); CORNACEAE (121/41); CORYNOCARPACEAE (4/1); COSTACEAE (591/38); CRASSULACEAE (151/38); CRUCIFERAE (778/360); CUCURBITACEAE (2175/247); CUNONIACEAE (473/36); CUPRESSACEAE (169/84); CUSCUTACEAE (105/23); CYCADACEAE (6/10); CYCLANTHACEAE (256/30); CYMODOCEACEAE (3/3); CYPERACEAE (9590/1154); CYRILLACEAE (33/6); DIALYPETALANTHACEAE (25/2); DICHAPETALACEAE (293/44); DICLIDANTHERACEAE (22/7); DILLENIACEAE (2238/108); DIOSCOREACEAE (2179/150); DIPSACACEAE (45/28); DIPTEROCARPACEAE (12/33); DRACAENACEAE (29/12); DROSERACEAE (256/47); DUCKEODENDRACEAE (9/2); EBENACEAE (742/86); ELAEAGNACEAE (18/8); ELAEOCARPACEAE (991/98); ELATINACEAE (6/5); EMPETRACEAE (5/2); EPACRIDACEAE (28/22); EPHEDRACEAE (32/16); ERICACEAE (2111/298); ERIOCAULACEAE (2816/464); ERYTHROXYLACEAE (3067/163); ESCALLONIACEAE (77/8); EUPHORBIACEAE (16907/1973); EUPHRONIACEAE (4/3); FABACEAE (57/14); FAGACEAE (176/134); FLACOURTIACEAE (4123/204); GELSEMIACEAE (16/5); GENTIANACEAE (2239/250); GERANIACEAE (207/91); GESNERIACEAE (2849/284); GINKGOACEAE (2/1); GNETACEAE (112/13); GOODENIACEAE (56/19); GOUPIACEAE (9/3); GRISELINIACEAE (14/2); GUNNERACEAE (18/7); GUTTIFERAE (3053/379); HAEMODORACEAE (57/12); HALORAGACEAE (62/18); HAMAMELIDACEAE (17/14); HELICONIACEAE (1420/100); HEMEROCALLIDACEAE (18/10); HERNANDIACEAE (140/18); HIPPOCASTANACEAE (10/9); HIPPOCRATEACEAE (1133/113); HIPPURIDACEAE (2/1); HUMIRIACEAE (1237/82); HYACINTHACEAE (29/20); HYDNORACEAE (1/1); HYDROCHARITACEAE (108/27); HYDROLEACEAE (24/5); HYDROPHYLLACEAE (103/38); HYPERICACEAE (505/61); HYPOXIDACEAE (106/10); ICACINACEAE (688/64); ILLECEBRACEAE (5/4); IRIDACEAE (1336/243); IXONANTHACEAE (3/2); JUGLANDACEAE (24/31); JUNCACEAE (386/110); JUNCAGINACEAE (19/5); KRAMERIACEAE (165/10); LACISTEMATACEAE (625/17); LAMIACEAE (6952/907); LARDIZABALACEAE (2/2); LAURACEAE (12630/753); LECYTHIDACEAE (2281/214); LEGUMINOSAE (78391/7103); LENTIBULARIACEAE (889/91); LEPIDOBOTRYACEAE (2/2); LILIACEAE (164/75); LINACEAE (206/50); LINDERNIACEAE (9/5); LISSOCARPACEAE (8/2); LOASACEAE (166/46); LOBELIACEAE (8/3); LOGANIACEAE (1495/152); LOMANDRACEAE (2/2); LORANTHACEAE (5629/455); LYTHRACEAE (2867/300); MAESACEAE (11/2); MAGNOLIACEAE (173/66); MALESHERBIACEAE (4/4); MALPIGHIACEAE (11646/706); MALVACEAE (7095/846); MARANTACEAE (3594/275); MARCGRAVIACEAE (707/45); MARTYNIACEAE (13/9); MAYACACEAE (109/8); MELANTHIACEAE (29/14); MELASTOMATACEAE (30923/2316); MELIACEAE (4466/272); MENISPERMACEAE (1316/130); MENYANTHACEAE (113/10); MOLLUGINACEAE (205/12); MONIMIACEAE (4887/158); MONTIACEAE (2/1); MORACEAE (8940/799); MORINGACEAE (11/5); MUNTINGIACEAE (19/2); MUSACEAE (38/23); MYOPORACEAE (26/15); MYRICACEAE (33/17); MYRISTICACEAE (1545/119); MYRTACEAE (25903/1808); NARTHECIACEAE (1/1); NELUMBONACEAE (1/2); NEPENTHACEAE (32/31); NYCTAGINACEAE (3302/127); NYMPHAEACEAE (227/46); OCHNACEAE (3076/252); OLACACEAE (1331/127); OLEACEAE (334/121); ONAGRACEAE (1854/201); OPILIACEAE (229/13); ORCHIDACEAE (13096/2258); OROBANCHACEAE (239/57); OXALIDACEAE (1366/159); PANDANACEAE (16/16); PAPAVERACEAE (233/99); PASSIFLORACEAE (2958/203); PEDALIACEAE (15/9); PELLICIERACEAE (2/2); PENTAPHYLACACEAE (42/12); PERACEAE (94/15); PERIDISCACEAE (7/2); PHILESIACEAE (4/3); PHYLLANTHACEAE (231/54); PHYTOLACCACEAE (992/54); PICRAMNIACEAE (137/19); PICRODENDRACEAE (3/1); PINACEAE (73/100); PIPERACEAE (18265/631); PITTOSPORACEAE (57/22); PLANTAGINACEAE (451/106); PLATANACEAE (15/8); PLUMBAGINACEAE (218/55); POACEAE (18370/2588); PODOCARPACEAE (187/29); PODOSTEMACEAE (114/43); POLEMONIACEAE (128/68); POLYGALACEAE (6825/458); POLYGONACEAE (2373/256); PONTEDERIACEAE (461/38); PORTULACACEAE (556/41); POTAMOGETONACEAE (93/46); PRIMULACEAE (4897/332); PROTEACEAE (945/146); PUNICACEAE (21/3); PUTRANJIVACEAE (80/12); PYROLACEAE (24/13); QUIINACEAE (374/51); QUILLAJACEAE (2/1); RAFFLESIACEAE (65/9); RANUNCULACEAE (1031/376); RAPATEACEAE (312/60); RESEDACEAE (9/4); RESTIONACEAE (9/9); RHABDODENDRACEAE (4/5); RHAMNACEAE (1643/176); RHIZOPHORACEAE (185/26); ROSACEAE (1930/596); RUBIACEAE (31966/3124); RUPPIACEAE (35/6); RUSCACEAE (13/7); RUTACEAE (3974/476); SABIACEAE (110/21); SACCIFOLIACEAE (2/1); SALICACEAE (1272/188); SALVADORACEAE (2/2); SANTALACEAE (260/67); SAPINDACEAE (8942/519); SAPOTACEAE (4937/524); SARRACENIACEAE (17/14); SAURURACEAE (5/4); SAXIFRAGACEAE (388/133); SCHEUZERIACEAE (2/2); SCHISANDRACEAE (2/2); SCHLEGELIACEAE (41/12); SCHOEPFIACEAE (18/3); SCROPHULARIACEAE (2936/605); SIMAROUBACEAE (961/103); SIPARUNACEAE (232/20); SMILACACEAE (3730/79); SOLANACEAE (14488/1256); SONNERATIACEAE (2/3); SPHENOCLEACEAE (3/1); STACHYURACEAE (4/1); STAPHYLEACEAE (29/11); STEMONURACEAE (17/2); STERCULIACEAE (2398/268); STILBACEAE (2/2); STRELITZIACEAE (12/7); STYRACACEAE (630/52); SURIANACEAE (6/1); SYMPLOCACEAE (757/66); TACCACEAE (11/7); TAMARICACEAE (28/14); TAXACEAE (13/8); TEPUIANTHACEAE (8/5); THEACEAE (646/66); THURNIACEAE (10/2); THYMELAEACEAE (562/100); TILIACEAE (1518/146); TOFIELDIACEAE (10/5); TRAPACEAE (4/2); TRIGONIACEAE (961/45); TRIURIDACEAE (32/13); TROPAEOLACEAE (60/17); TURNERACEAE (1120/155); TYPHACEAE (111/14); ULMACEAE (1009/88); URTICACEAE (2916/232); VALERIANACEAE (237/193); VELLOZIACEAE (1471/206); VERBENACEAE (5550/541); VIOLACEAE (2500/256); VITACEAE (1266/123); VIVIANIACEAE (23/11); VOCHYSIACEAE (3971/237); WINTERACEAE (242/17); XANTHORRHOEACEAE (35/26); XYRIDACEAE (1135/181); ZAMIACEAE (19/15); ZANNICHELLIACEAE (8/2); ZINGIBERACEAE (532/63); ZOSTERACEAE (3/2); ZYGOPHYLLACEAE (92/46).

**Group**: Ferns & Lycophytes

**Family**: ANEMIACEAE (607 specimens/58 names); ASPLENIACEAE (2532/183); ATHYRIACEAE (716/109); BLECHNACEAE (1340/99); CULCITACEAE (17/4); CYATHEACEAE (1329/162); CYSTOPTERIDACEAE (48/14); DAVALLIACEAE (56/32); DENNSTAEDTIACEAE (502/100); DESMOPHLEBIACEAE (2/1); DICKSONIACEAE (145/27); DIPTERIDACEAE (4/2); DRYOPTERIDACEAE (3708/578); EQUISETACEAE (142/20); GLEICHENIACEAE (664/59); HEMIDICTYACEAE (28/1); HYMENOPHYLLACEAE (2099/170); ISOETACEAE (39/16); LINDSAEACEAE (539/74); LOMARIOPSIDACEAE (377/46); LYCOPODIACEAE (1620/167); LYGODIACEAE (375/15); MARATTIACEAE (198/24); MARSILEACEAE (57/17); MATONIACEAE (3/1); METAXYACEAE (62/4); OLEANDRACEAE (50/12); ONOCLEACEAE (8/3); OPHIOGLOSSACEAE (83/17); OSMUNDACEAE (115/19); PLAGIOGYRIACEAE (27/4); POLYPODIACEAE (5733/589); PSILOTACEAE (33/3); PTERIDACEAE (5280/577); SACCOLOMATACEAE (110/8); SALVINIACEAE (233/18); SCHIZAEACEAE (1524/69); SELAGINELLACEAE (1294/133); TECTARIACEAE (262/23); THELYPTERIDACEAE (1529/237); WOODSIACEAE (51/16).

**Group**: Bryophytes

**Family**: ACROBOLBACEAE (3 specimens/3 names); ADELANTHACEAE (7/5); ADELOTHECIACEAE (7/1); AMBLYSTEGIACEAE (87/25); ANDREAEACEAE (43/13); ANEURACEAE (255/19); ANOMODONTACEAE (11/8); ANTHOCEROTACEAE (45/8); ARNELLIACEAE (25/1); AULACOMNIACEAE (8/3); AYTONIACEAE (11/4); BALANTIOPSIDACEAE (142/12); BARTRAMIACEAE (202/61); BRACHYTHECIACEAE (386/68); BRUCHIACEAE (12/4); BRYACEAE (318/60); CALLIERGONACEAE (5/4); CALYMPERACEAE (475/46); CALYPOGEIACEAE (53/12); CATAGONIACEAE (6/2); CEPHALOZIACEAE (45/18); CEPHALOZIELLACEAE (57/15); CHONECOLEACEAE (12/3); CORSINIACEAE (3/3); CRYPHAEACEAE (15/10); DALTONIACEAE (8/5); DENDROCEROTACEAE (23/4); DICRANACEAE (357/88); DITRICHACEAE (40/26); DUMORTIERACEAE (85/2); ENTODONTACEAE (37/12); EPHEMERACEAE (1/1); ERPODIACEAE (40/6); EUSTICHIACEAE (7/5); FABRONIACEAE (24/9); FISSIDENTACEAE (270/59); FONTINALACEAE (18/10); FOSSOMBRONIACEAE (24/5); FRULLANIACEAE (635/43); FUNARIACEAE (50/19); GEOCALYCACEAE (48/15); GRIMMIACEAE (73/47); GYMNOMITRIACEAE (15/7); HEDWIGIACEAE (31/9); HELICOPHYLLACEAE (21/1); HERBERTACEAE (73/14); HOOKERIACEAE (54/28); HYDROPOGONACEAE (3/1); HYLOCOMIACEAE (19/8); HYPNACEAE (2/64); HYPNACEAE (327/64); HYPOPTERYGIACEAE (85/12); JAMESONIELLACEAE (121/13); JUNGERMANNIACEAE (75/32); LEJEUNEACEAE (1/255); LEJEUNEACEAE (2439/255); LEMBOPHYLLACEAE (153/25); LEPICOLEACEAE (6/2); LEPIDOZIACEAE (361/58); LEPYRODONTACEAE (7/1); LESKEACEAE (25/12); LEUCOBRYACEAE (622/89); LEUCODONTACEAE (17/8); LEUCOMIACEAE (8/3); LOPHOCOLEACEAE (202/29); LUNULARIACEAE (10/1); MARCHANTIACEAE (134/7); METEORIACEAE (131/37); METZGERIACEAE (613/53); MNIACEAE (96/32); MONOCLEACEAE (48/2); MYRINIACEAE (2/2); NECKERACEAE (250/48); NOTOTHYLADACEAE (15/4); ORTHODONTIACEAE (11/3); ORTHOTRICHACEAE (351/83); PALLAVICINIACEAE (259/13); PELLIACEAE (40/5); PHYLLODREPANIACEAE (1/1); PHYLLOGONIACEAE (98/6); PHYMATOCEROTACEAE (1/1); PILOTRICHACEAE (444/70); PLAGIOCHILACEAE (615/65); PLAGIOTHECIACEAE (15/6); PLEUROZIACEAE (1/1); POLYTRICHACEAE (1/46); POLYTRICHACEAE (346/46); PORELLACEAE (45/9); POTTIACEAE (471/167); PRIONODONTACEAE (33/7); PTERIGYNANDRACEAE (2/2); PTEROBRYACEAE (65/16); PTYCHOMITRIACEAE (25/7); PTYCHOMNIACEAE (1/1); PYLAISIADELPHACEAE (96/16); RACOPILACEAE (63/3); RADULACEAE (243/28); RHABDOWEISIACEAE (63/16); RHACHITHECIACEAE (3/3); RHACOCARPACEAE (64/5); RHIZOGONIACEAE (141/11); RICCIACEAE (52/22); RIGODIACEAE (21/2); RUTENBERGIACEAE (2/1); SCAPANIACEAE (56/22); SELIGERIACEAE (2/2); SEMATOPHYLLACEAE (427/52); SPHAGNACEAE (1/114); SPHAGNACEAE (707/114); SPLACHNACEAE (5/4); STEREOPHYLLACEAE (57/7); SYMPHYODONTACEAE (2/1); THUIDIACEAE (172/35); TRICHOCOLEACEAE (43/8).

**Group**: Algae

**Family**: ACINETOSPORACEAE (40 specimens/10 names); ANADYOMENACEAE (33/6); ARESCHOUGIACEAE (3/3); BANGIACEAE (99/28); BONNEMAISONIACEAE (39/8); BOODLEACEAE (20/2); BRYOPSIDACEAE (159/32); CALLITHAMNIACEAE (187/71); CAULERPACEAE (295/34); CERAMIACEAE (516/104); CHAMPIACEAE (101/22); CHARACEAE (103/44); CHLAMYDOMONADACEAE (28/21); CHNOOSPORACEAE (5/1); CHORDARIACEAE (199/76); CLADOPHORACEAE (835/163); CLOSTERIACEAE (64/31); CODIACEAE (259/17); CORALLINACEAE (3/65); CORALLINACEAE (484/65); CYSTOCLONIACEAE (279/22); DASYACEAE (163/40); DASYCLADACEAE (9/4); DELESSERIACEAE (186/62); DERBESIACEAE (20/3); DESMIDIACEAE (273/193); DICTYOTACEAE (620/85); DUMONTIACEAE (11/9); ECTOCARPACEAE (75/35); ERYTHROTRICHIACEAE (3/2); GALAXAURACEAE (97/12); GELIDIACEAE (204/31); GELIDIELLACEAE (43/7); GIGARTINACEAE (174/32); GRACILARIACEAE (296/30); HALIMEDACEAE (41/8); HALYMENIACEAE (216/31); HILDENBRANDIACEAE (5/3); HYPNEACEAE (74/5); KALLYMENIACEAE (27/9); KORNMANNIACEAE (6/2); LAMINARIACEAE (57/18); LIAGORACEAE (22/14); LOMENTARIACEAE (79/18); NEMASTOMATACEAE (4/3); PALMOPHYLLACEAE (3/1); PEYSSONNELIACEAE (18/7); PHYLLOPHORACEAE (113/14); PLOCAMIACEAE (74/9); POLYPHYSACEAE (49/7); PTEROCLADIACEAE (169/5); PYLAIELLACEAE (18/3); RHIZOPHYLLIDACEAE (15/2); RHODOMELACEAE (1334/278); RHODYMENIACEAE (146/38); SARGASSACEAE (411/58); SCENEDESMACEAE (16/11); SCHIZYMENIACEAE (3/3); SCINAIACEAE (5/3); SCYTOSIPHONACEAE (71/17); SEBDENIACEAE (5/1); SIPHONOCLADACEAE (45/9); SOLIERIACEAE (66/17); SPHACELARIACEAE (38/17); SPOROCHNACEAE (27/13); SPYRIDIACEAE (44/16); UDOTEACEAE (39/15); ULVACEAE (1/58); ULVACEAE (587/58); VALONIACEAE (15/10); WRANGELIACEAE (118/42); ZYGNEMATACEAE (199/110).

## Collection data

### Collection name

Herbário Dimitri Sucre Benjamin

### Collection identifier

RB

### Specimen preservation method

Dried and pressed

## Usage rights

### Use license

Other

### IP rights notes

This work is licensed under a Creative Commons Attribution 4.0 International License.

## Data resources

### Data package title

RB - Rio de Janeiro Botanical Garden Herbarium Collection

### Resource link

http://www.gbif.org/dataset/4300f8d5-1ae5-49e5-a101-63894b005868

### Number of data sets

1

### Data set 1.

#### Data set name

RB - Rio de Janeiro Botanical Garden Herbarium Collection

#### Data format

Darwin Core Archive (DwC-A)

#### Number of columns

46

#### Download URL

http://ipt.jbrj.gov.br/jbrj/archive.do?r=jbrj_rb&v=84.131

#### Description

The RB herbarium has 750,000 mounted specimens, making it the largest herbarium in Brazil (Gasper and Vieira 2015). The full database is available via the Integrated Publishing Toolkit (IPT) of Rio de Janeiro Botanical Garden (Version 84.131, published in 2017-11-22) or via JABOT system (http://www.jbrj.gov.br/jabot). For the integration of Web-based applications, the JABOT Web service is available at http://servicos.jbrj.gov.br/jabot.

**Data set 1.** 

Column label	Column description
occurrenceID	The unique identifier of the Occurrence.
identifiedBy	A list (concatenated and separated) of names of people, groups or organisations who assigned the Taxon to the subject.
dateIdentified	The date on which the subject was identified as representing the Taxon.
identificationRemarks	Comments or notes about the identification.
identificationQualifier	A standard term ("cf.", "aff.") to express the determiner's doubts about the identification.
typeStatus	Status of the type. Controlled vocabulary of terms (HOLOTIPO, LECTOTIPO, ISOTIPO, SINTIPO, PARATIPO, NEOTIPO, EPITIPO, TYPUS). The category "TYPUS" is used for undefined type status.
scientificName	The full scientific name, with authorship.
family	The full scientific name of the family in which the taxon is classified.
genus	The full scientific name of the genus in which the taxon is classified.
SpecificEpithet	The name of the first or species epithet of the scientificName.
infraspecificEpithet	The name of the lowest or terminal infraspecific epithet of the scientificName.
taxonRank	The taxonomic rank of the most specific name in the scientificName.
scientificNameAuthorship	The authorship information for the scientificName.
format	Image file format.
identifier	A list, concatenated and separated by "|" of the specimens images URLs in a low-resolution format to be used as thumbnails.
references	A list, concatenated and separated by "|" of the specimens images URLs in a high-resolution format to be integrated into other portals and websites.
license	A legal document giving official permission to do something with the resource.
rightsHolder	A person or organisation owning or managing rights over the resource.
type	The nature or genre of the resource.
modified	The most recent date-time on which the resource was changed.
institutionCode	The name (or acronym) in use by the institution having custody of the object(s) or information referred to in the record.
collectionCode	The name, acronym, coden or initialism identifying the collection or data set from which the record was derived.
basisOfRecord	The specific nature of the data record.
catalogNumber	Barcode of the specimen.
recordNumber	The collector's number.
recordedBy	A list (concatenated and separated) of names of people, groups or organisations responsible for recording the original occurrence.
otherCatalogNumbers	Sequential register number historically adopted by the RB herbarium.
associatedMedia	A list, concatenated and separated by "|" of the specimens images URLs in a low-resolution format to be used as thumbnails. The content and sequence of this column is the same of the "License" column on the Multimedia extension and can be used as identifiers for the independent images.
eventDate	Date of collection.
year	Year of collection.
month	Month of collection.
day	Day of collection.
fieldNumber	An identifier given to the event in the field. Can be described as the number of the field campaign.
fieldNotes	The text of notes taken in the field about the specimen.
eventRemarks	Comments or notes about the field campaign.
country	The name of the country or major administrative unit in which the Location occurs.
countryCode	The standard code for the country in which the Location occurs according to ISO 3166-1-alpha-2 country codes.
stateProvince	The name of the next smaller administrative region than country (state, province, canton, department, region etc.) in which the Location occurs.
county	The full, unabbreviated name of the next smaller administrative region than stateProvince (county, shire, department etc.) in which the Location occurs.
locality	The specific description of the place. Less specific geographic information can be provided in other geographic terms (higherGeography, continent, country, stateProvince, county, municipality, waterBody, island, islandGroup). This term may contain information modified from the original to correct perceived errors or to standardise the description.
minimumElevationInMeters	The lower limit of the range of elevation (altitude, usually above sea level), in metres.
maximumElevationInMeters	The upper limit of the range of elevation (altitude, usually above sea level), in metres.
verbatimLatitude	The verbatim original latitude of the Location.
verbatimLongitude	The verbatim original longitude of the Location.
decimalLatitude	The geographic latitude (in decimal degrees, using the spatial reference system given in geodeticDatum) of the geographic centre of a Location. Positive values are north of the Equator, negative values are south of it. Legal values lie between -90 and 90, inclusive.
decimalLongitude	The geographic longitude (in decimal degrees, using the spatial reference system given in geodeticDatum) of the geographic centre of a Location. Positive values are east of the Greenwich Meridian, negative values are west of it. Legal values lie between -180 and 180, inclusive.

## Additional information

### Recent projects

**Project title**: “Informatização do Herbário do Jardim Botânico do Rio de Janeiro” (Digitisation of the herbarium of the Rio de Janeiro Botanical Garden).

**Project description**: The digitisation of specimens started with the creation of an institutional system, known as JABOT. Constant upgrades were made during the last decade, including the construction of a digitisation module and improvements to the public interface.

**Funding**: Petrobras (Petróleo Brasileiro S.A.)

**Duration**: 2005-2007

**Project title**: Global Plants Initiative

**Project description**: The project focused on constructing a database and producing high-resolution images (600 dpi) of all type specimens deposited at RB. The image capture workflow involved inverted scanners developed by the Royal Botanic Gardens, Kew, called HerbScan.

**Funding**: The Andrew W. Mellon Foundation

**Duration**: 2007-2011

**Project title**: “Plantas do Brasil: Resgate Histórico e Herbário Virtual para o conhecimento e conservação da flora brasileira – REFLORA” (Plants of Brazil: Historical Rescue and a Virtual Herbarium for knowledge and conservation of the Brazilian flora – REFLORA)

**Project description**: This project, coordinated by the JBRJ, developed a virtual herbarium for public access, including samples collected in the Brazilian territory, during the 18th, 19th and 20th centuries, deposited at European herbaria. Concomitantly with the repatriation process, this project also financed the imaging of the RB specimens, which were also published on the Reflora Virtual Herbarium.

**Funding**: Brazilian National Council for Scientific and Technological Development (CNPq); Research Support Foundation of the State of Rio de Janeiro (FAPERJ); Minas Gerais State Agency for Research and Development (FAPEMIG); Vale Foundation, Natura Cosmetics and Newton Fund.

**Duration**: 2010-2016

**Project title**: “Conhecimento e conservação da flora brasileira: futuros desafios das coleções biológicas do Jardim Botânico do Rio de Janeiro” (Knowledge and conservation of the Brazilian flora: Future challenges for the biological collections of the Rio de Janeiro Botanical Garden).

**Project description**: The project, aimed to capture data and images of RB specimens previously unavailable in the JABOT system, especially those in the fungi, bryophyte, algae, fruits and wood collections. It also facilitated visits by taxonomists, improving the quality of identifications, particularly for those groups for which there are no specialists at the RB.

**Funding**: The Brazilian Science, Technology and Innovation Ministry (MCTI), CNPq and The Brazilian Biodiversity Information System (SiBBr).

**Duration**: 2012-2015

**Project title**: “Contribuições do Jardim Botânico do Rio de Janeiro à implementação do SiBBr” (Contributions of the Rio de Janeiro Botanical Garden to the implementation of the SiBBr).

**Project description**: The Brazilian Biodiversity Information System (SiBBr), is an initiative that intends to ensure the proper use of Brazilian biodiversity and ecosystem data, integrating information and facilitating processes of decision-making and public policy development. It also assists national herbaria in the digitisation of their specimens and in the repatriation of images of specimens from European and North American herbaria. The RB contributes to this initiative by making available its collections' data and those of Reflora Virtual Herbarium and the Brazilian Flora 2020, projects coordinated by this institution. This initiative currently supports daily data and image capture of new specimens incorporated into RB herbarium.

**Funding**: MCTI, SiBBr, CNPq

**Duration**: 2014-2017

**Project title**: “Inventário Florestal Nacional” (The National Forest Inventory)

**Project description**: The National Forestry Inventory (IFN) is coordinated by the Brazilian Forest Service (SFB) and aims to collect socio-economic and ecological information about the country's forest resources. It supports the formulation, implementation and execution of public policies for the development, use and conservation of these resources. In the state of Rio de Janeiro, the process for identification of botanical material has been carried out by taxonomist consultants hired by the IFN in the RB. In addition, all the fertile specimens collected by the project in the other Brazilian states are also sent to the RB. This project financed the acquisition of imaging equipment for all participant herbaria, contributing to collections digitisation. Also, at RB, due to its size, three technicians were hired to help with day-to-day herbarium activities.

**Funding**: Brazilian Forest Service

**Duration**: 2013-current

### Challenges for biological collections digitisation and publication

The costs related to the maintenance and curation of a herbarium are significant and curators are always under pressure to gain financial support (Deng 2015). There is also a demand for modernisation and data sharing, which greatly increases the costs for these collections, augmenting the need for financial support. Those new costs come mainly from IT infrastructure and its maintenance but also originate from a demand for new and specialised staff and for software maintenance and development.

The first phases of the project of digitisation and publishing the contents of the RB herbarium occurred between 2005 and 2007, with an investment of around US$254,000 for the incorporation of metadata of 291,630 specimens and digitisation of 10,646 specimens (Gonzales 2009). From 2007 to 2011, the Global Plants Initiative focused on the type specimens that are few in number comparing to the general collection. From 2010, a new phase of the digitisation of the RB herbarium started under the umbrella project Reflora, which has the following as one of its main goals: the repatriation of high resolution images of Brazilian specimens deposited in European and North American herbaria and the digitisation of many national herbaria. Considering the fact that this phase is included in such an enormous initiative, the quantification of the amount of investments expended exclusively with RB is imprecise, at least for the time being.

Regarding infrastructure, the dataset associated with the RB collection represents 2 5.6% of all institutional digital storage space, 6.8% of processing power (CPUs) and 9.7% of memory. The associated costs of power consumption, especially of climate control in the tropics, are also significant.

Despite the fact that literature cites a number of initiatives of online open-access biodiversity databases that failed due to lack of funding, after the initial push for resources (Costello et al. 2014), there has been a solid perception that nowadays "Without data we cannot generate information and build knowledge to make informed decisions or develop indicators to track progress towards biodiversity goals and targets" (Juffe-Bignoli et al. 2016).

Thus, it is considered that the trade-off for committing a substantial portion of theinstitutional budget, as well as technical and scientific staff time, to digitisation of and publication about the collections, has been very positive for the institutional relevance, as well as for its visibility and image and this is associated with a resulting gain in funding opportunities.
